# Augmented-Reality-Assisted K-Wire Placement for Glenoid Component Positioning in Reversed Shoulder Arthroplasty: A Proof-of-Concept Study

**DOI:** 10.3390/jpm11080777

**Published:** 2021-08-10

**Authors:** Klaus Schlueter-Brust, Johann Henckel, Faidon Katinakis, Christoph Buken, Jörg Opt-Eynde, Thorsten Pofahl, Ferdinando Rodriguez y Baena, Fabio Tatti

**Affiliations:** 1 Department of Orthopaedic Surgery, St. Franziskus Hospital Köln, 50825 Köln, Germany; Faidon-Ioannis.Katinakis@cellitinnen.de (F.K.); Christoph.Buken@cellitinnen.de (C.B.); info@opti3d.de (J.O.-E.); 2 Institute of Orthopaedics, The Royal National Orthopaedic Hospital, Brockley Hill, Stanmore, London HA7 4LP, UK; johann.henckel@nhs.net; 3 Demo Working Group GbR, 50676 Köln, Germany; pofahl@trako.arch.rwth-aachen.de; 4 Mechatronics in Medicine Laboratory, Imperial College London, London SW7 2AZ, UK; f.rodriguez@imperial.ac.uk (F.R.y.B.); f.tatti@imperial.ac.uk (F.T.)

**Keywords:** augmented reality, image-guided surgery, intraoperative imaging, simulation, mixed reality, reversed shoulder arthroplasty, 3D printing, 3D planning

## Abstract

The accuracy of the implant’s post-operative position and orientation in reverse shoulder arthroplasty is known to play a significant role in both clinical and functional outcomes. Whilst technologies such as navigation and robotics have demonstrated superior radiological outcomes in many fields of surgery, the impact of augmented reality (AR) assistance in the operating room is still unknown. Malposition of the glenoid component in shoulder arthroplasty is known to result in implant failure and early revision surgery. The use of AR has many promising advantages, including allowing the detailed study of patient-specific anatomy without the need for invasive procedures such as arthroscopy to interrogate the joint’s articular surface. In addition, this technology has the potential to assist surgeons intraoperatively in aiding the guidance of surgical tools. It offers the prospect of increased component placement accuracy, reduced surgical procedure time, and improved radiological and functional outcomes, without recourse to the use of large navigation or robotic instruments, with their associated high overhead costs. This feasibility study describes the surgical workflow from a standardised CT protocol, via 3D reconstruction, 3D planning, and use of a commercial AR headset, to AR-assisted k-wire placement. Post-operative outcome was measured using a high-resolution laser scanner on the patient-specific 3D printed bone. In this proof-of-concept study, the discrepancy between the planned and the achieved glenoid entry point and guide-wire orientation was approximately 3 mm with a mean angulation error of 5°.

## 1. Introduction

Early failure rates and sub-optimal performance continue to plague outcomes in reverse shoulder arthroplasty. Whilst the causes of revision surgery and poor function are multifactorial and include patient, implant factors, and surgeon, implant malposition remains a constant. Several computer-assisted strategies and tools are in use with varying outcomes.

Traditional instruments remain the mainstay for the preparation of the glenoid in reverse shoulder arthroplasty, and whilst there are sophisticated 3D planning systems available on the market, delivering these virtual plans remains a challenge even for experienced surgeons [1].

Although not as common as hip or knee arthroplasty, shoulder arthroplasty has become more widely adopted in recent years [2]. Reverse total shoulder arthroplasty (RTSA) is known to be an effective surgical procedure for glenohumeral arthritis, rotator cuff arthropathy, irreparable rotator cuff tears, complex proximal humerus fractures, and failed shoulder prosthesis [3].

The Norwegian Arthroplasty Register reports a 5-year survival rate of 90% for RTSA, a result similar to a 2006 multicentre study by Guery et al. [4,5]. Results from the Register reveal that aseptic loosening of the glenoid component is one of the main causes of early revision surgery. Implant loosening is often due to technical errors, such as the glenoid component being positioned too high and/or in superior inclination [6], which induces severe shear stress, impairing fixation [7,8]. Consequently, precise positioning of the glenoid component is crucial to avoid impingement and premature loosening, and to improve the survival rate [9].

Augmented reality (AR) can be a valuable tool to increase accuracy in both bone preparation and implant placement in surgery. In contrast to virtual reality, which creates a completely virtual environment to the exclusion of the real world, AR overlays virtual information onto a real environment, so that intuitive guidance is provided [10].

Among the various options available, optical-see-through head-mounted-displays (OST-HMD) are the preferrable choice for introducing AR in orthopaedic surgery, due to their flexibility and the fact that they allow a natural, unobstructed view of the scene when the AR is switched off [11]. In recent years, several commercial optical see-through products such as the Microsoft HoloLens (Microsoft, Redmond, WA, USA) and Google Glass (Google Inc., Mountain View, CA, USA) have become widely available.

A small number of solutions for AR-based intraoperative surgical guidance have been successfully demonstrated in humans, e.g., for spine [12] and hip [13] surgery. Nevertheless, AR has not been widely adopted and the vast majority of surgeries are still performed manually, without any computer-assisted aids.

To the best of our knowledge, no such solutions exist yet for shoulder arthroplasty, and only one previous study [14] has demonstrated the use of augmented reality for assisted placement of the glenoid component in Total Shoulder Arthroplasty (TSA). This paper presents a proof-of-concept system to provide AR guidance during k-wire placement for glenoid component positioning in reversed shoulder arthroplasty, using the Microsoft HoloLens 2 system. The system was trialled on 3D-printed scapula phantoms derived from real patient anatomy, and the k-wire entry point and orientational errors are reported.

## 2. Materials and Methods

### 2.1. Imaging Data

A single CT scan of an osteoarthritic right shoulder was used as reference anatomy for the study. The scan was obtained using the BLUEPRINT™ CT protocol [15], from a 78-year-old female patient with 29.2 BMI, diagnosed with rotator cuff arthropathy, who qualified for reverse shoulder arthroplasty. The scan was completed using a Canon Aquilion 64 scanner, with 0.5 mm collimation width. To improve the image quality and optimise segmentation outcome, a pillow was inserted between the patient’s arm and body, to distract the humerus head from the glenoid. The position of the arm was stabilised using a strap. The study had internal institution review board (IRB) approval together with informed consent of the patient. The CT DICOM data were anonymised following standard data protection protocols.

### 2.2. Procedure Planning

The DICOM CT scan files were imported into the mediCAD^®^ 3D Shoulder software (mediCAD Hectec GmbH, Altdorf/Landshut, Germany) and segmented using an automated procedure provided by the software, followed by manual refinement. A 3D model of the scapula was then reconstructed from the segmented slices.

Surgical planning was performed by loading the CAD model of the implant’s glenoid component into the mediCAD software and manually adjusting its position relative to the patient anatomy, to achieve optimal placement. Tornier Aequalis™ Perform™ Reversed implants (Wright Medical Group, Memphis, TN, USA) were used for this study. A 2.5 mm guidance k-wire model was then loaded into the software and positioned using the implant post as reference.

The reconstructed 3D models with and without the planned k-wire position were exported in STL format for use in the subsequent steps of the study.

### 2.3. Procedure Execution

One experienced shoulder arthroplasty surgeon performed all of the procedures. To avoid learning effects, data analysis was performed after all procedures had been completed, and the surgeon was unaware of the outcome of completed trials when performing subsequent ones.

**Figure 1:**
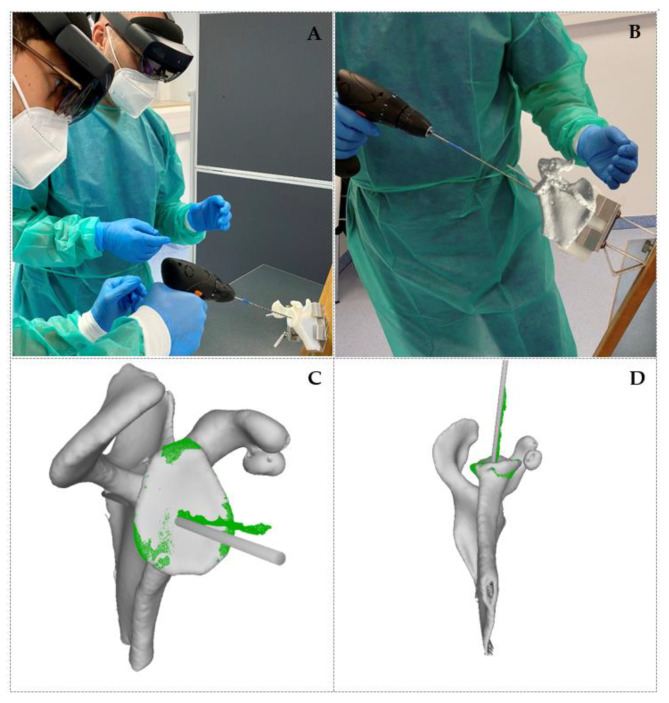
(**A**) Surgical setup and demonstration of the k-wire insertion. (**B**) View of the surgical scene from the HoloLens 2 device, with the AR reference overlaid onto the phantom scapula. (**C**,**D**) Three-quarter and bottom views of a 3D-scanned phantom (green) registered to the reference anatomy with planned k-wire position (grey). The 3D scan is cropped to include only the glenoid, to optimise registration quality in this area.

Nine phantom models of the scapula were produced by 3D printing the exported STL file using a Stratasys Polyjet 3D printer (Stratasys, Eden Prairy, MN, USA). Conventional bone clamps were used to support the phantoms, which were placed in simulated beach chair position during execution of the procedure, as shown in Figure 1A.

AR guidance was provided via a Microsoft HoloLens 2 device worn by the surgeon. The STL model including the planned k-wire position was loaded onto the HoloLens 2 and holographically displayed in front of the surgeon (see Figure 1B), via the mediCAD^®^ MR App (Beta Version). The position of the virtual anatomical model was manually adjusted to match that of the 3D-printed phantom.

Finally, the surgeon inserted a k-wire into the 3D-printed phantom with a standard drill, using the position of the virtual k-wire as a reference.

### 2.4. Error Measurement

After the k-wire was inserted, the 3D-printed phantoms with the k-wire were digitised using a professional, high-resolution 3D scanner (Artec Space Spyder, Artec3D, Luxembourg).

The model obtained from the 3D scanner was imported into the Blender software (Blender Foundation, Amsterdam, The Netherlands) and co-registered with the preoperative surgical plan by first coarsely aligning the scapula models manually, and then refining the alignment using the iterative closest point (ICP) method [16]. In order to optimise the alignment of the glenoid, points from other regions of the scapula were excluded from the ICP routine (see Figure 1C,D). Once the models were aligned, the orientational error between the planned and achieved k-wire positions was measured by fitting a cylinder to the points corresponding to the planned insertion and scanned drill, respectively, then recording the angular distance between the two as reported by Blender’s angular measurement tool. Subsequently, the entry point error was measured by identifying the intersection between the two cylinders and the glenoid surface, and recording their distance, as reported by Blender’s distance measurement tool.

## 3. Results

### 3.1. Qualitative Results

The addition of preoperative 3D procedure planning increased the overall time required by approximately 5 min. This preoperative stage, however, provided extremely valuable 3D information, with better anatomical orientation, better visualisation, and the possibility to obtain a 3D-printed haptic patient-specific model, for consenting the patient, examining the anatomy, and practicing the surgery. Furthermore, the 3D data could be valuable for surgical education and training.

During the intraoperative stage, both the AR headset and the mediCAD^®^ MR App proved intuitive and easy to use. The headset was comfortable to wear and did not induce any fatigue. The use of AR increased the time required for k-wire insertion by about 3 min. The additional time was primarily required for the manual alignment of the holographic reference anatomy to the phantom scapula.

While inserting the k-wire, it was crucial to minimize head movements in order to maintain optimal alignment between the holographic reference and the phantom. This limited the surgeon’s comfort during this stage of the operation, and was highlighted as a challenge to be addressed in order to successfully introduce the technology in an operating theatre.

### 3.2. Quantitative Results

To evaluate the registration error between the 3D-scanned scapulas and the reference anatomy, we measured the distance of each point in the glenoid region of the scans (green area in Figure 1C,D) to the corresponding nearest neighbour on the reference anatomy. The average distance was around 0.5 mm for all the scapulas, indicating good alignment.

**Figure 2:**
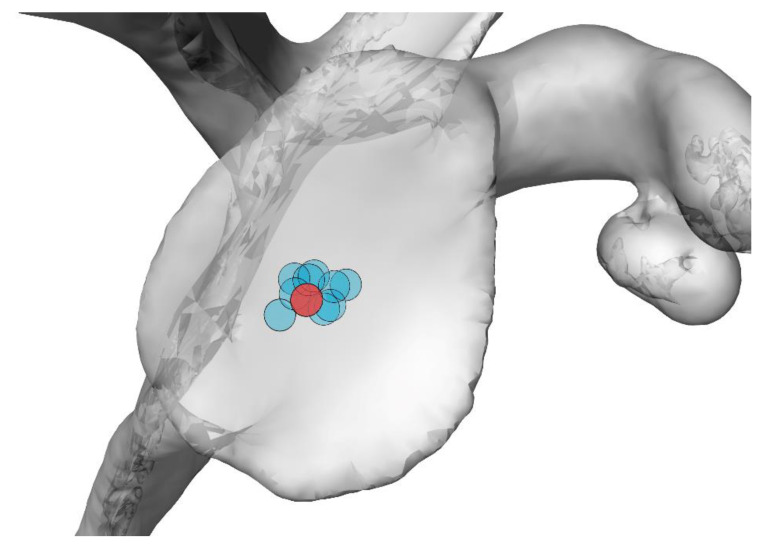
Graphical illustration of the actual entry point for all trials (light blue) relative to the planned entry point (red).

The measured errors between the planned and achieved entry point and k-wire orientation are reported in Table 1, for all the phantoms in the same order in which they were tested. The same results are illustrated graphically in Figure 2. The average ± sd entry-point error was 2.4 ± 0.7 mm, while the average ± sd orientational error was 3.9 ± 2.4°. Table 1 does not highlight evidence of a learning effect.

## 4. Discussion

Our lab results in this proof-of-concept study compare favourably with published data in conventional surgery [17].

The majority of published articles using standard instrumentation techniques reported mean postoperative version errors of 7.1° (min. 3.5° to max. 11.2°), mean postoperative inclination errors of 8.45° (min. 2.8° to max. 11.65°) and mean postoperative positional offset errors of 2.6 mm (min. 1.7 mm to max. 3.4 mm) compared with preoperative plans [17].

The entry-point accuracy measured in this study is comparable to a previously published study demonstrating the use of the HoloLens 1 headset for glenoid component placement, which reported an average entry-point error of 2.3 mm [14]. The study, however, reported a lower average orientational error (2.7°), which might be explained by the use of an automated registration method based on surface scanning of the glenoid.

We have demonstrated the feasibility of replicating the pre-operative CT-based plan in this lab-based study. The use of the high-resolution laser scanner introduced minimal noise to the measurement of the discrepancy between the planned and achieved position and orientation of the guide wire.

Real challenges in the clinical application of this technology in the context of image registration include the presence of residual cartilage on the articular surface in a CT-based planning system. Increased surgical dissection and access are needed and the incision will need to remain distracted to maintain the initial registration until the guide wire is inserted. Blood and residual soft tissues can also obscure the field of view.

Whilst the execution of the plan was the primary objective in this exercise, the challenges of pre-operative surgical planning must not go unmentioned. The quality of the CT scan, including satisfactory distraction of the worn articular surfaces, is needed to facilitate optimal bone segmentation. It can be difficult to assess the bone quality and hence decide upon the ideal position in which to plan and seat the glenoid component. However, the limitations described here are ubiquitous to all image-based Computer-Assisted Surgery (CAS) systems, including Patient-Specific Instrumentation (PSI) jigs, navigation systems, and potential robotic assisted solutions.

Patient specific jig systems are becoming more widely adopted, with many different design strategies in clinical use with varying degrees of radiological measured accuracy. Cabarcas’s [17] systematic review of PSI-guided surgery reveals that the results of our pilot study compare favourably with their use.

Because of the increased operative experience of the several senior surgeons who were involved in these above-cited studies, the implant position error was low in comparison to low-volume surgeons. However, the mean errors in our experiment were superior (average ± sd entry-point error was 2.4 ± 0.7 mm, while the average ± sd orientational error was 3.9 ± 2.4°) to those in these studies using standard instrumentation techniques.

Our results cannot be compared directly to studies using PSI for shoulder arthroplasty, but the reported mean postoperative version and inclination errors of 5° or less compared with preoperative plans [17,18] are equal to our results, which were obtained simply using a see-through device (HoloLens 2). This finding suggests that AR-based aid can be particularly advantageous for novice or low-volume shoulder surgeons.

Particular challenges with PSI include the more extensive surgical dissection in order to gain access to seat and secure the bespoke jig to the glenoid. The need for increased surgical releases increases the risk of neuro-vascular injury. Additional challenges in the positioning of the jigs include the presence of unworn cartilage on the periarticular edges of the glenoid leading to poor seating of the guide and the potential for malorientation of the guide wire trajectory. PSI remains a viable option in assisting the surgeon to deliver his/her plan and are relatively inexpensive, costing around 400 € per case.

Computer navigation systems are also in clinical use. They offer greater accuracy and precision in guide wire placement; these systems are relatively expensive, however, in comparison to the results from our AR concept demonstrator, their results are comparable.

Accurate intraoperative landmark registration remains a challenge with greater surgical access needed. Line-of-sight issues with tracking continue to confront the user [19].

Robotic systems for the shoulder are not yet in clinical use; however, the large implant companies are planning to expand into shoulder arthroplasty. They promise sub-millimetre implant accuracy; however, they are very expensive and those in use in hip and knee arthroplasty can cost in excess of 1 million €.

From a technical point of view, the primary challenge that needs to be addressed in order to for AR to become a viable tool for surgery is the accuracy of the calibration between the virtual content displayed by the headset and the real scene. In this proof-of-concept study the operating surgeon was required to align the virtual hologram to the target anatomy manually. Aside from being a laborious operation, the manual alignment is also highly subjective and prone to human error. Future research will therefore look at the incorporation of automated methods for the registration of virtual content onto the target anatomy.

Various methods have been proposed in the literature to automatically align virtual content to a target anatomy. A highly accurate overlay of virtual content was demonstrated in [20], using fiducial markers and a custom head-mounted display. The authors reported an error below 1 mm in a maxillofacial surgical task conducted on plastic bone. While marker-based tracking is an established technology that provides great accuracy, and it is currently the norm for computer-assisted surgical navigation, there are disadvantages to its use in arthroplasty. Indeed, the need to use pins to rigidly attach trackers might increase the risk of complications [21]. In order to remove the need for rigidly attached markers, some groups have developed markerless computer-vision-based solutions that rely on the registration between a preoperative model of the patient anatomy and a 3D model of the same anatomy obtained intraoperatively, either by scanning the surface with a probe (e.g., [14]), or by exploiting the onboard sensors available on the headset (e.g., [10]). While the accuracy of these solutions is currently inferior to marker-based tracking, it is a rapidly expanding research field that holds great promise.

While knowledge of the relative position between the headset and the surgical site is necessary to obtain good alignment, it is not sufficient. Indeed, it is also necessary to account for the optics of the headset and its interaction with the user’s eyes. Commercially available general-purpose optical-see-through systems use simple calibration methods whose accuracy is not sufficient for use in surgery, and they generally display the AR content at a fixed focal distance, which introduces perceptual issues for surgical tasks in the peripersonal space. Research in this topic is ongoing, and several methods have been proposed (e.g., [22,23]) to increase the accuracy of general-purpose headsets currently available on the market. Additionally, various companies and research groups have worked on the development of bespoke head-mounted displays, specifically tailored to the needs of intraoperative surgical guidance, e.g., [20,24].

## 5. Conclusions

The promise of augmented reality to overlay 3D virtual information onto a real scene has vast potential for orthopaedic surgery. AR is, however, a novel technology, still in its infancy, and a number of technical challenges still need to be addressed before it can be considered viable for use in clinical practice. The fast pace at which AR technology is moving and the amount of research interest that it is attracting make us hopeful that AR systems with the required specifications will be available in the future, and that this technology will become part of clinical practice.

## Figures and Tables

**Table 1:** Entry point and orientation errors for all phantoms tested.

**Phantom ID**	1	2	3	4	5	6	7	8	9
**Entry point (mm)**	2.8	1.9	1.2	1.8	2.3	2.8	2.3	3.9	2.3
**Orientation (°)**	9.0	5.3	6.7	2.3	2.1	1.8	4.2	2.2	1.7

## Data Availability

All data are available upon request.
